# Post-cardiac injury syndrome triggered by radiofrequency ablation for AVNRT

**DOI:** 10.1186/s12872-021-02436-1

**Published:** 2021-12-25

**Authors:** Florian A. Wenzl, Martin Manninger, Stefanie Wunsch, Daniel Scherr, Egbert H. Bisping

**Affiliations:** 1 Department of Cardiology, Medical University of Graz, Graz, Austria; 2 Center for Molecular Cardiology, University of Zurich, Zurich, Switzerland; 3 Section of Infectious Diseases and Tropical Medicine, Department of Internal Medicine, Medical University of Graz, Graz, Austria

**Keywords:** Radiofrequency ablation, Inflammation, AVNRT, Post-cardiac injury syndrome

## Abstract

**Background:**

Post-cardiac injury syndrome (PCIS) is an inflammatory condition following myocardial or pericardial damage. In response to catheter ablation, PCIS most frequently occurs after extensive radiofrequency (RF) ablation of large areas of atrial myocardium. Minor myocardial injury from right septal slow pathway ablation for atrioventricular nodal reentrant tachycardia (AVNRT) is not an established cause of the syndrome.

**Case presentation:**

A 62-year-old women with a 6-year history of symptomatic narrow-complex tachycardia was referred to perform an electrophysiological study. During the procedure AVNRT was recorded and a total of two RF burns were applied to the region between the coronary sinus and the tricuspid annulus. Pericardial effusion was routinely ruled out by focused cardiac ultrasound. In the following days, the patient developed fever, elevated inflammatory and cardiac markers, new-onset pericardial effusion, characteristic ECG changes, and complained of pleuritic chest pain. An extensive workup for infectious, metabolic, rheumatologic, neoplastic, and toxic causes of pericarditis and myocarditis was unremarkable. Cardiac magnetic resonance imaging showed no signs of ischemia, infiltrative disease or structural abnormalities. The patient was diagnosed with PCIS and initiated on aspirin and low-dose colchicine. At a 1-month follow-up visit the patient was free of symptoms but still had a small pericardial effusion. After three  months of treatment the pericardial effusion had resolved completely.

**Conclusions:**

Inflammatory pericardial reactions can occur after minor myocardial damage from RF ablation without involvement of structures in close proximity to the pericardium.

## Background

Post-cardiac injury syndrome (PCIS) is an inflammatory reaction to myo-pericardial damage and constitutes a rare but important complication of radiofrequency (RF) catheter ablation [1, 2]. Serious clinical sequelae include massive pericardial effusion with delayed cardiac tamponade, pleural effusion, and hypoxemia [2]. Most frequently, PCIS is reported after ablation of large areas of atrial myocardium in close proximity to the pericardium [2–6]. Here, we describe a unique case of PCIS after right septal ablation for atrioventricular nodal reentrant tachycardia (AVNRT).

## Case presentation

**Fig. 1:**
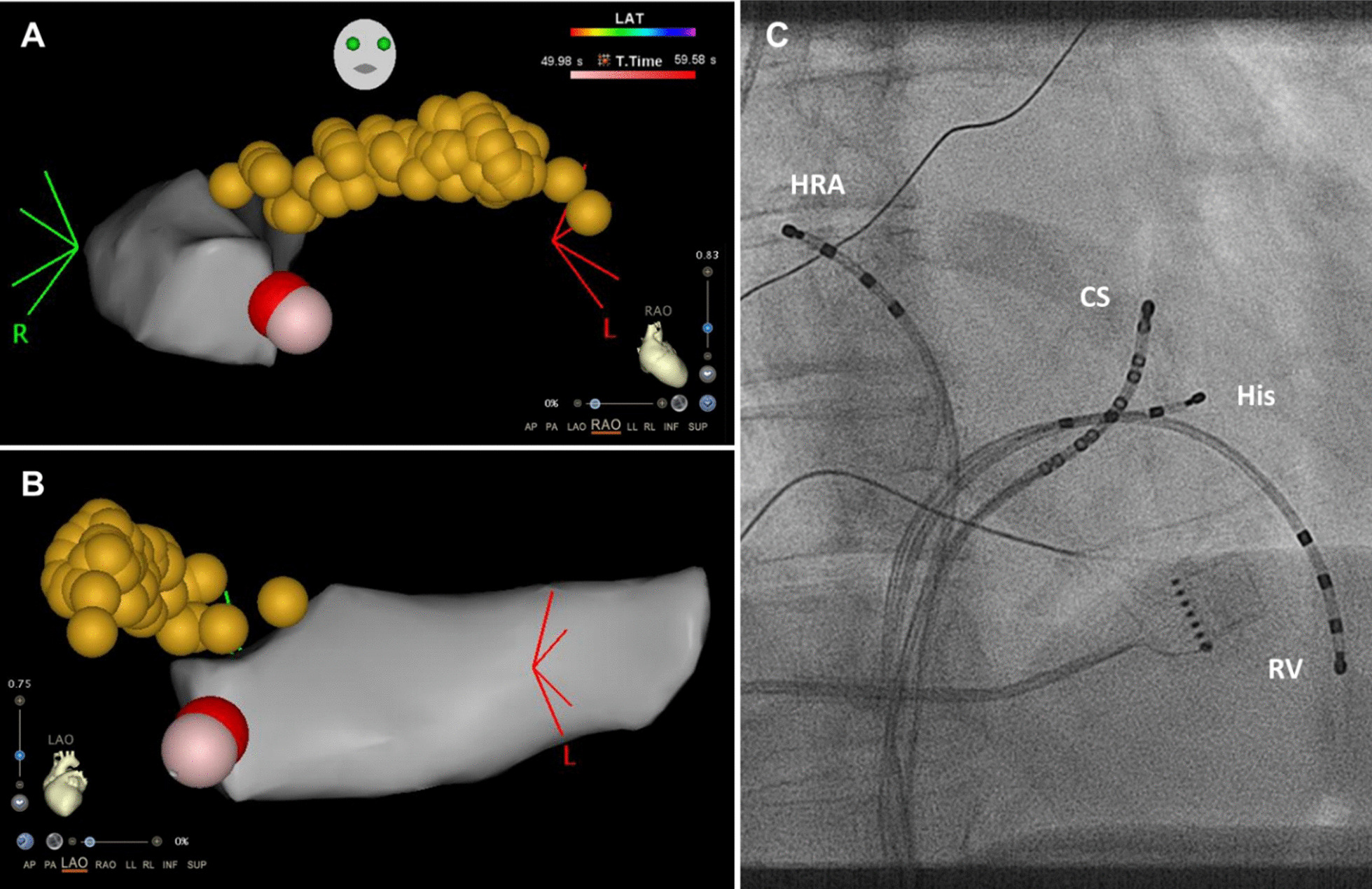
Region of energy application and catheter position during the procedure. Electroanatomic map showing the RF burns (purple and red), the coronary sinus (grey), and the His bundle region (orange) in right (**A**) and left (**B**) anterior oblique view. Appropriate position of the catheter tips (**C**) in the high right atrium (HRA), in the coronary sinus (CS), at the His region (His), and at the right ventricular apex (RV)

**Fig. 2:**
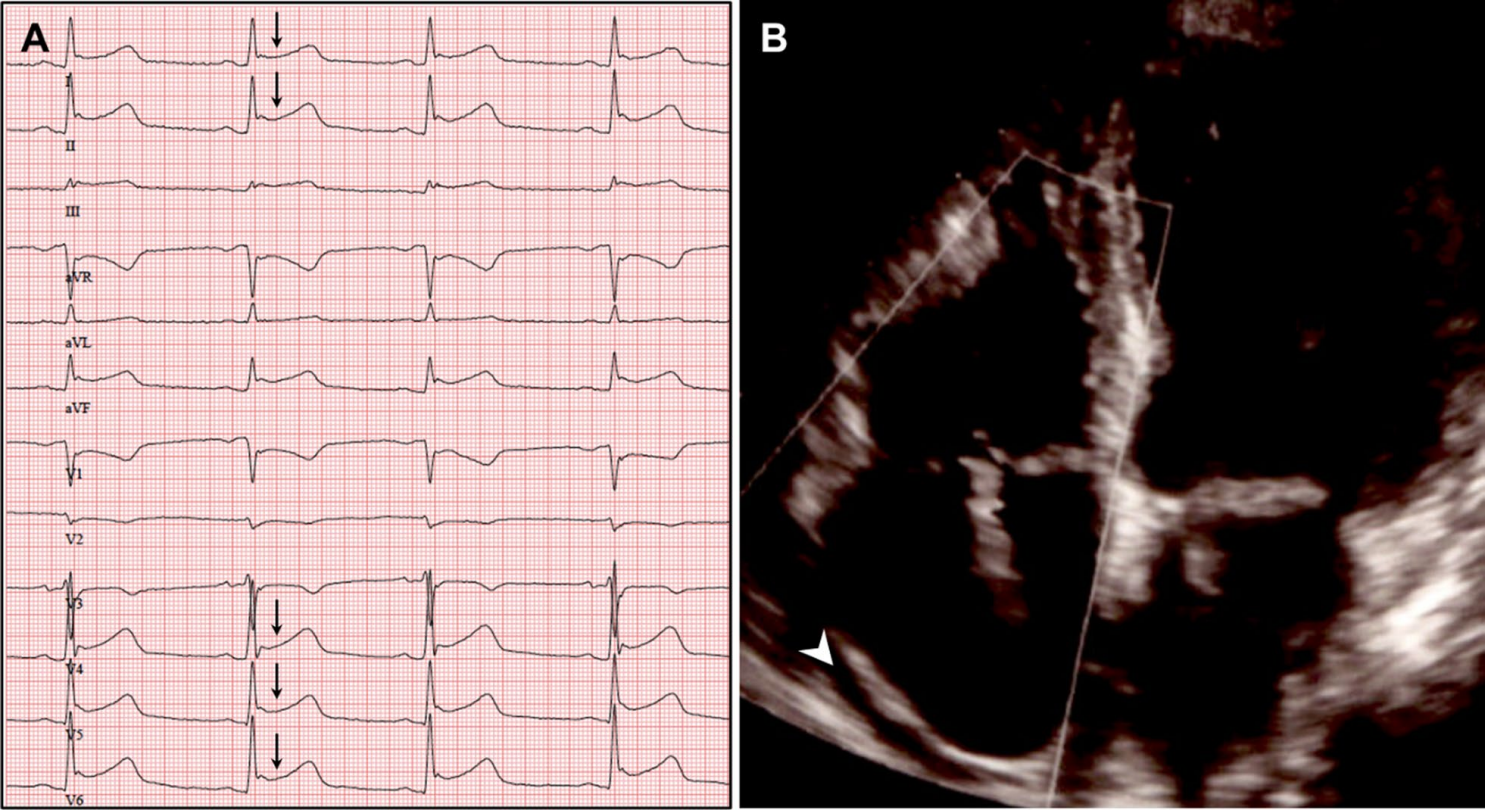
New-onset ECG changes and pericardial effusion. 12-lead ECG showing ST segment elevations in leads I, II, aVL, and V_4_–V_6_ three days after ablation (**A**). New-onset circumferential pericardial effusion detected on transthoracic echocardiography (**B**)

A 62-year-old female with a 6-year history of paroxysmal narrow-complex tachycardia was referred for an electrophysiologic study. On admission, the patient was asymptomatic and her routine clinical assessment and physical examination were unremarkable. During her electrophysiology study, sustained typical AVNRT was induced and RF ablation was performed with a non-irrigated Navistar ablation catheter (Biosense Webster) using three-dimensional electroanatomic guidance. A total of two RF burns were applied to the region between the coronary sinus and tricuspid annulus. The first RF burn (35 W [W], 12 Ohm impedance drop during 50 s) showed good junction response but more than 2 echo beats persisted after the energy application. A second burn (35 W, 11 Ohm impedance drop during 60 s) resulted in successful slow pathway ablation (Fig. 1). Pericardial effusion was ruled out by postprocedural focused cardiac ultrasound which is routinely performed at our centre. During the next days, the patient developed a low-grade fever of 38 degrees Celsius, mild tachycardia, leucocytosis, elevated cardiac markers, and complained of chest pain on inspiration. Creatinine kinase (CK) was elevated at 168 U/L and high sensitivity cardiac Troponin T at 294 pg/mL. A new-onset circumferential pericardial effusion was detected on echocardiography and the electrocardiogram (ECG) showed diffuse saddle-shaped ST segment elevations (Fig. 2). An extensive workup for infectious, metabolic, rheumatologic, neoplastic, and toxic causes of pericarditis and myocarditis was unremarkable. Gadolinium-enhanced cardiac magnetic resonance imaging showed no signs of ischemia, infiltrative disease or structural abnormalities. According to current clinical practice guidelines [1] a diagnosis of post-cardiac injury syndrome (PCIS) was made and the patient was started on aspirin and low-dose colchicine. Within three days of treatment, symptoms and ECG changes resolved, the pericardial effusion was reduced, and the patient could be discharged home. One month later, the patient still showed a small pericardial effusion and was continued on aspirin and colchicine. At a 3-month follow-up, the patient was doing well and the pericardial effusion had resolved completely.

## Discussion

PCIS is an umbrella term for a variety of inflammatory pericardial reactions following damage to the myocardium or pericardium. PCIS was first described by Dressler [7] and is characterized by fever, pleuritic chest pain, pericardial effusion and elevated inflammatory markers [1]. Such inflammatory syndromes are presumed to have an autoimmunologic pathogenesis which is supported by their tendency for recurrence [1, 8]. Aside from extensive injury secondary to myocardial infarction or open surgery involving pericardiotomy, PCIS can be caused by minimal cardiac damage from pacemaker lead insertion [9], transcatheter mitral valve repair [10], transcatheter aortic valve replacement [10], and percutaneous coronary intervention [11] in predisposed individuals. Overall, PCIS develops in up to 1-5% of percutaneous cardiac interventions [12].

In response to catheter ablation, PCIS most frequently occurs after extensive energy application during ablation of large areas of left or right atrial myocardium in close intimacy to the visceral pericardium [2]. According to a report by Li et al. [2], over 70% of cases due to catheter ablation are caused by left atrial ablation for atrial fibrillation and right atrial ablation for atrial flutter [2]. Interestingly, in our patient the syndrome occurred secondary to a very minor trauma by only two RF burns without involvement of structures in proximity to the pericardium supporting the concept of an underlying immunologic reaction. The pathophysiology of PCIS after catheter ablation is poorly understood [2], yet putative triggers may include myocardial necrosis from the RF burn leading to the release of auto-antigens [1, 2], mechanical trauma to the pericardium during the procedure, and intraprocedural tachycardia in combination with an underlying substrate, such as a subclinical myocarditis. While differential diagnoses, including infectious, metabolic, rheumatologic, neoplastic, and toxic aetiologies, were ruled out in an extensive clinical workup, we acknowledge that, in order to minimize patient risk, no confirmatory histopathological analyses of myocardial or pericardial specimens were conducted. A markedly shorter delay to symptom onset in patients with PCIS after catheter ablation [2–6, 13–15] compared to other cardiac injuries suggests different underlying mechanisms and distinguishes this entity from others within the spectrum of PCIS. In line with the literature [2–6, 13–15], our patient developed PCIS within the first week after the intervention and responded well to medical treatment with aspirin and low-dose colchicine.

Treatment of PCIS is based on anti-inflammatory agents including aspirin, non-steroidal anti-phlogistic drugs, corticosteroids, and colchicine [1, 16]. Aspirin is recommended as a first-line treatment of PCIS while colchicine may be considered as an add-on therapy [1]. Timely detection and medical therapy of PCIS commonly leads to complete resolution of signs and symptoms and is key to preventing long-term sequelae such as recurrent pericardial effusion and pericardial constriction [1, 12]. Given the increasing use of catheter ablation for the treatment of cardiac arrythmia, awareness of potential complications is critical to guide patient care.

## Conclusions

Inflammatory pericardial reactions can be triggered by minor myocardial injury from right septal slow pathway ablation in susceptible individuals. Clinical surveillance in the postprocedural period helps to identify complications.

## Data Availability

All relevant data supporting the conclusions of this article are included in the article. The datasets used and/or analysed during the current study are available from the corresponding author on reasonable request.

## Abbreviations

ANRT: Atrioventricular nodal reentrant tachycardia
CK: Creatinine kinase
CS: Coronary sinus
His: His region
ECG: Electrocardiogram
HRA: High right atrium
PCIS: Post-cardiac injury syndrome
PE: Pericardial effusion
RF: Radiofrequency
RV: Right ventricular apex
W: Watt
